# Local and systemic effect of transfection-reagent formulated DNA vectors on equine melanoma

**DOI:** 10.1186/s12917-015-0422-9

**Published:** 2015-06-11

**Authors:** Kathrin Mählmann, Karsten Feige, Christiane Juhls, Anne Endmann, Hans-Joachim Schuberth, Detlef Oswald, Maren Hellige, Marcus Doherr, Jessika-MV Cavalleri

**Affiliations:** Clinic for Horses, University of Veterinary Medicine Hannover, Foundation, Hannover, Germany; Mologen AG, Berlin, Germany; Immunology Unit, University of Veterinary Medicine Hannover, Foundation, Hannover, Germany; Institute for Veterinary Epidemiology and Biostatistics, Free University of Berlin, Berlin, Germany

**Keywords:** Horse, Melanoma, Interleukin, Glycoprotein 100, Tyrosinase, DNA vaccine

## Abstract

**Background:**

Equine melanoma has a high incidence in grey horses. Xenogenic DNA vaccination may represent a promising therapeutic approach against equine melanoma as it successfully induced an immunological response in other species suffering from melanoma and in healthy horses. In a clinical study, twenty-seven, grey, melanoma-bearing, horses were assigned to three groups (n = 9) and vaccinated on days 1, 22, and 78 with DNA vectors encoding for equine (eq) IL-12 and IL-18 alone or in combination with either human glycoprotein (hgp) 100 or human tyrosinase (htyr). Horses were vaccinated intramuscularly, and one selected melanoma was locally treated by intradermal peritumoral injection. Prior to each injection and on day 120, the sizes of up to nine melanoma lesions per horse were measured by caliper and ultrasound. Specific serum antibodies against hgp100 and htyr were measured using cell based flow-cytometric assays. An Analysis of Variance (ANOVA) for repeated measurements was performed to identify statistically significant influences on the relative tumor volume. For post-hoc testing a Tukey-Kramer Multiple-Comparison Test was performed to compare the relative volumes on the different examination days. An ANOVA for repeated measurements was performed to analyse changes in body temperature over time. A one-way ANOVA was used to evaluate differences in body temperature between the groups. A p–value < 0.05 was considered significant for all statistical tests applied.

**Results:**

In all groups, the relative tumor volume decreased significantly to 79.1 ± 26.91% by day 120 (p < 0.0001, Tukey-Kramer Multiple-Comparison Test). Affiliation to treatment group, local treatment and examination modality had no significant influence on the results (ANOVA for repeated measurements). Neither a cellular nor a humoral immune response directed against htyr or hgp100 was detected. Horses had an increased body temperature on the day after vaccination.

**Conclusions:**

This is the first clinical report on a systemic effect against equine melanoma following treatment with DNA vectors encoding eqIL12 and eqIL18 and formulated with a transfection reagent. Addition of DNA vectors encoding hgp100 respectively htyr did not potentiate this effect.

**Electronic supplementary material:**

The online version of this article (doi:10.1186/s12917-015-0422-9) contains supplementary material, which is available to authorized users.

## Background

Equine melanoma, a tumor of pigment producing cells, is the most common skin tumor in aging grey horses with a prevalence of up to 95% [1].

So far, conventional therapies such as surgical excision [2], cryosurgery [2], radiotherapy [3], or chemotherapy with cisplatin [4] or cimetidine [5] have not been curative in advanced cases. Obviously, there is need for innovative approaches to treat equine melanoma lesions of later stages.

Xenogenic DNA vaccination against the melanoma differentiation antigens glycoprotein (gp) 100 [6-9] and tyrosinase (tyr) [10-13] have been shown to overcome auto-tolerance and to elicit an immune response in mice, dogs, and humans [10-16] and a clinical antitumoral effect in mice and dogs [10-12,14,16].

In clinically healthy horses, specific antibodies at relatively low levels and a variable cellular immune response were elicited upon vaccination with a plasmid encoding the human tyrosinase (htyr) [17]. There are no reports about the immunogenicity of human gp100 (hgp100) to horses, and clinical study results on the anti-melanoma effect of DNA-encoded xenogenic tyrosinase and gp100 in grey horses have not been published to date.

Cytokines such as Interleukin (IL) 12 and IL18 have been applied to increase cellular immunity and reduce angiogenesis in neoplasms [18-23] and were shown to have synergistic antitumoral effects [20,24,25,26].

To date, all immunological melanoma treatment efforts in horses were of limited effect. A local effect was achieved with DNA encoding human [27] or either eqIL12 or eqIL18 [28]. Thus, combination of these interleukins and addition of either hgp100 or htyr, all encoded by DNA vectors, seemed to be a promising approach.

The aim of the present clinical study was to evaluate whether or not treatment with eqIL12 in combination with eqIL18 each encoded by DNA vectors has a local and systemic anti-tumoral effect on naturally occurring melanoma in grey horses, and whether or not this effect is augmented by DNA vaccination against the xenogenic melanoma differentiation antigens hgp100 and htyr, respectively.

## Methods

### Patients

Twenty-seven horses with one or more melanoma lesions and unaffected general condition were included in the study. Informed consent was obtained from all animal owners. Horses were not treated with any medication at least two weeks prior to immunisation.

Pre-trial evaluation included a physical examination, hematology, and blood biochemistry profile. Age, breed, gender, degree of greying and number of melanoma lesions were documented (Table 1). The diagnosis of melanoma was confirmed by examination of fine needle aspirates by board certified pathologists in 20 of 27 horses. In the remaining horses, diagnosis was made clinically with regard to typical localisation and appearance of the lesions, as the owners did not agree to aspiration biopsy. Patients were treated in the Clinic for Horses of the University of Veterinary Medicine Hannover, Foundation, from November 2009 to July 2010 in accordance with the ethical guidelines of the law of animal welfare approved by the “Lower Saxony State Office for Consumer Protection and Food Safety, LAVES” approval No. 08/1522).

**Table 1:** **Patient demographics and group assignment**

**Treatment (encoded genes)**	**Horse ID**	**Age (years)**	**Breed**	**Sex**	**Color**	**Number of Melanoma**	**FNA**	**Localisation**
eqIL12, eqIL18	1	22	Warmblood	m	flea bitten	>7	no	vt, mu, o
	2	18	Warmblood	g	flea bitten	1	yes	vt
	7	20	Icelandic-Horse	m	white	>7	yes	pa, vt, ge
	9	12	Berber	m	white	>7	yes	pa, vt, ge, p
	12	11	Carmargue	m	dappled	>7	no	vt, pa, ge, p
	13	11	Shetland	g	dappled	3	no	pa, ge, vt
	17	17	Andalusian	m	white	>7	yes	pa, vt, ge, mu, o
	23	14	Arabian	g	flea-bitten	1	yes	p
	24	16	Warmblood	m	flea-bitten	>7	yes	pa, vt, ge
eqIL12, eqIL18, hgp100	3	18	unclassified	g	flea bitten	1	yes	pa
	5	12	Andalusian	m	dappled	>7	yes	pa, vt, mu, p, o
	6	18	Andalusian	s	white	>7	no	e, vt, ge
	8	15	Berber	s	white	>7	yes	pa, vt
	15	14	Andalusian	s	white	>7	no	pa, vt
	18	16	Warmblood	m	white	>7	yes	pa, vt, ge
	19	15	Arabian	g	flea-bitten	>7	yes	p
	22	14	Warmblood	m	flea-bitten	2	no	pa, vt
	27	22	Icelandic-Horse	m	flea-bitten	>7	yes	pa, vt, ge
eqIL12, eqIL18, htyr	4	19	Arabian	s	flea bitten	>7	yes	pa, vt
	10	15	Arabian	s	white	>7	yes	pa, vt, mu
	11	20	Carmargue	s	white	4	no	vt
	14	15	Shetland	m	white	>7	no	vt
	16	13	Warmblood	m	flea-bitten	2	yes	pa, vt
	20	22	Trakehner	g	white	>7	yes	pa, vt
	21	19	Arabian	g	white	1	yes	vt
	25	13	Arabian	m	white	>7	yes	pa, vt, ge, mu, o
	26	11	Irish horse (Hunter)	g	white	1	yes	vt
**Legend:**								
m: mare			vt: ventral tail		e: eyelid			
g: gelding			p: parotid region		mu: muscle			
s: stallion			ge: genitals		o: other regions			
FNA: fine needle aspirate			pa: perianal					

Horses were assigned to three treatment groups by a stratified biphasic model to achieve equal distribution of age (<15 years or ≥ 15 years) and number of melanoma lesions (<7 or ≥ 7 melanoma lesions).

### Production of MIDGE-Th1 vectors

Minimalistic immunogenically defined gene expression (MIDGE) vectors with a small peptide (“Th1”) attached were produced by MOLOGEN AG (Berlin, Germany) as previously described [29] from plasmids coding for hgp 100 (pcDNA3gp100, courtesy of Dr. Robbins und Dr. Rosenberg, National Cancer Institute), htyr (pMCV1.4htyr, MOLOGEN AG), eqIL12 and eqIL18 (pUSErIRESeqIL12 and 18, courtesy of L. Nicholson, University of Glasgow, via Intervet International, Boxmeer, The Netherlands). Oligonucleotides coding for the IL-1beta receptor antagonist protein (ILRAP) (Microsynth, Balgach, Switzerland) were ligated to the IL18 gene (see Additional file 1: Appendix A).

### Preparation of MIDGE-Th1/SAINT-18 complexes

MIDGE-Th1/SAINT-18 (1-methyl-4-(cis-9-dioleyl) methyl-pyridinium-chloride, Synvolux Therapeutics, Groningen, The Netherlands) complexes were prepared as described by Endmann et al. [30]. MIDGE-Th1/SAINT-18 complexes were formed at a ratio of 1 mg DNA dissolved in PBS to 0.75 μmol SAINT-18. The PBS concentration in the final mixture was 1× PBS.

### Treatment

Three groups of 9 grey horses each were treated on days 1, 22 and 78 with MIDGE-Th1 vectors coding for eqIL12 and ILRAP-eqIL18 alone or in combination with hgp100MIDGE-Th1 or htyrMIDGE-Th1 (Table 2). Throughout the study period the test items were blinded by a color code and unblinded upon completion of data analysis only.

**Table 2:** **Treatment substances administered to horses of the three treatment groups on days 1, 22 and 78**

**Vaccine component**	**Dose per injection**		
	**Group eqIL12/18**	**Group hgp100**	**Group htyr**
MIDGE-Th1	200 μg	200 μg	200 μg
eqIL12			
MIDGE-Th1	200 μg	200 μg	200 μg
eqILRAPIL18			
MIDGE-Th1	-	500 μg	-
hgp100			
MIDGE-Th1	-	-	500 μg
htyr			
SAINT 18	0.35 μmol	0.675 μmol	0.675 μmol

For each treatment, 0.5 ml (half of the vaccine dose) were injected intradermally (i.d.) peritumorally around one pre-selected, well-measureable and easily-accessible melanoma (“locally treated melanoma”). Injection was performed using a 25 G cannula and a 1 ml syringe. Another 0.5 ml (the second half of the dose) were administered intramuscularly (i.m.) into the semimembranosus muscle using a 22 G cannula and a 1 ml syringe. The identical injection sites were used for the first, second and third immunisation.

### Clinical evaluation for determination of response to treatment

In each horse the locally treated melanoma and up to eight additional melanoma lesions (“non-locally treated melanoma lesions”) were monitored prior to each injection and on day 120 using calipers and ultrasound (LogiQ P5, General Electrics, Connecticut, USA). The ultrasonographic measurements were performed independently by two examiners. All measurements were performed in triplicates. The length (mm, longest diameter), width (mm, perpendicular to length), and depth (mm, only by ultrasound) were documented and the tumor volume (cm^3^) calculated (volume = length x width^2^ x 0.5 for caliper measurements and volume = length x width x depth x 0.5 for ultrasonographic measurements).

The relative volumes were calculated in reference to the volume on day one (pre-dosing), which was defined as 100 %. For all recorded non–locally treated melanoma lesions of each horse, a mean value per time point was calculated to statistically evaluate the effect on these tumors, i.e. the systemic effect of the treatment. Furthermore, the change of tumor volume was calculated by subtraction of the relative volume on day 1 (100%) from the relative volume on day 120 (%), with a negative value implying tumor reduction and a positive value standing for tumor growth.

These measurements and calculations were used in previous studies investigating the effect of gene therapies against equine melanoma [28] and were found to be appropriate for this mostly slow growing tumor and for comparability with aforementioned studies.

### Clinical evaluation of safety of treatment

Horses were hospitalized for 3 days after each injection. Safety and tolerability of the treatment were evaluated by clinical examination, hematologic and blood biochemistry profile: a general clinical examination was performed before injection, on each day of hospitalisation and on day 120. A hematologic examination was realized before injection, on the 3rd day after injection and on day 120, a biochemistry profile was accomplished before each injection and on day 120. The injection sites were monitored for signs of local inflammation and depigmentation. Other pigmented skin areas (eyelids, nostrils) were observed for signs of depigmentation.

### Measurement of the immune response induced by vaccination

To measure specific serum antibodies against hgp100 and htyr a cell based, flow cytometric antibody assay was performed. Therefore, sera from the patients were obtained before each injection and on day 120. The sera were incubated with hgp100 and htyr plasmid transfected HEK 293 cells. Evaluation was performed by flow cytometry after staining bounded antibodies with a secondary antibody (see Additional file 1: Appendix A).

At any time post-vaccination a humoral response was considered positive when fluorescence increased ≥ 3 standard deviations over the mean value at baseline and had an absolute value > 0.1 %.

The establishment of an assay to detect a potential T-cell response induced by the vaccine was not followed up after transfection rates of primary autologous dermal cells, derived from skin of patients were inconsistent and low. Thus expression of antigens was insufficient to induce T cell activation after co-culture of transfected cells and peripheral blood mononuclear cells (data not shown).

### *In vitro* expression of transgenes eqIL12, eqIL18, htyr and hgp100 on mRNA level

Chinese hamster ovary (CHO)-K1 cells (ATCC CCL-61) were cultured in Ham’s F12 (10% FCS, 1% Penicillin/Streptomycin) medium at 37 °C in 5% CO_2_. Prior to transfection the culture medium was removed, cells were washed once with PBS, then detached with trypsin/EDTA and 0.12E + 06 cells per well suspended in 500 μL transfection medium (Ham’s F12 cell culture medium w/o additives). 100 μL of each DNA/SAINT-18 complex were prepared as follows: MIDGE-Th1 vectors were mixed with previously vortexed SAINT-18 (0.75 mM) at a ratio of 5 μl SAINT-18 per μg DNA and filled up to 100 μL with HBS. Complexes were allowed to form for 5 min. Cells were incubated with complexes containing a) MIDGE-Th1 vectors encoding eqIL12 and eqIL18 (0.5 μg per vector), b) MIDGE-Th1 vectors encoding eqIL12 (0.5 μg), eqIL18 (0.5 μg) and htyr (1.25 μg), c) MIDGE-Th1 vectors encoding eqIL12 (0.5 μg), eqIL18 (0.5 μg) and hgp100 (1.25 μg) and d) MIDGE-Th1 vectors encoding eGFP (1.25 μg) as positive control for the transfection method and CHO-K1 expression efficiency (measured by FACS). Salmon sperm DNA (Invitrogen) served as negative control item. The DNA SAINT-18 complexes were added to the cells, followed by a brief centrifugation step. After 2.5 hours of incubation, 1 mL of complete Ham’s F12 was added and cells incubated for 24 hours at 37 °C in 5% CO_2_. Cells were harvested and detached as described above, centrifuged and pellets stored on ice until RNA was extracted using the NucleoSpin RNA II Kit (Macherey & Nagel) as described in provider’s instructions.

mRNA specific Reverse Transcription quantitative PCR (RT-qPCR) was performed with 100 ng mRNA per reaction using the TaqMan® RNA-to-CT 1-Step Kit (Applied Biosystems) according to manufacturer’s instructions. Primers and probes (TIBMOLBIOL, Berlin) had specific sequences to generate and detect cDNA of eqIL12-p35 (fw 5’-AAATTGCTAACGCAGTCAGT-3’, rv 5’-GCTAGCTCCGGAGTT-3’, probe FAM-CGACTGATCACAGGGGTACC-BBQ), eqIL12-p40 (fw 5’-AAATTGCTAACGCAGTCAGT-3’, rv 5’-GACCAACCACTGGTGAC-3’, probe FAM-CGACTGATCACAGGGGTACC-BBQ), eqIL18 (fw 5’-AAATTGCTAACGCAGTCAGT-3’, rv 5’-GAGGCCTCTGCAGATT-3’, probe FAM-CGACTGATCACAGGGGTACC_BBQ), hgp100 (fw 5’-AAATTGCTAACGCAGTCAGT-3’, rv 5’-AGCCAAATGAAGAAGGCATC −3’, probe FAM-CGACTGATCACAGGGGTACC-BBQ) and htyr (fw 5’-AAATTGCTAACGCAGTCAGT-3’, rv 5’-CCACAGCAGGCAGTAC −3’, probe FAM-CGACTGATCACAGGGGTACC-BBQ). Samples were measured in technical triplicates.

### Statistical analysis

An Analysis of Variance (ANOVA) for repeated measurements was performed to identify statistically significant influences on the relative tumor volume. Parameters included in the model were the individual horse, examination day (1, 22, 78, 120), treatment group (eqIL12/18, hgp100, htyr), locally treated versus non-locally treated melanoma lesions and examination method (caliper and ultrasound with differentiation between the two examiners). After starting with the full model, non-significant variables and correlations were eliminated stepwise.

For post-hoc testing a Tukey-Kramer Multiple-Comparison Test was performed to compare the relative volumes on the different examination days.

An ANOVA for repeated measurements was performed to analyse changes in body temperature over time. A one-way ANOVA was used to evaluate differences in body temperature between the groups.

A p–value < 0.05 was considered significant for all statistical tests applied.

Statistical analyses were performed using the statistical software JMP 8.0 (SAS Institute Inc., Cary, NC, USA) and NCSS (NCSS, Kaysville, Utha, USA).

## Results

### Safety of treatment

**Figure 1:**
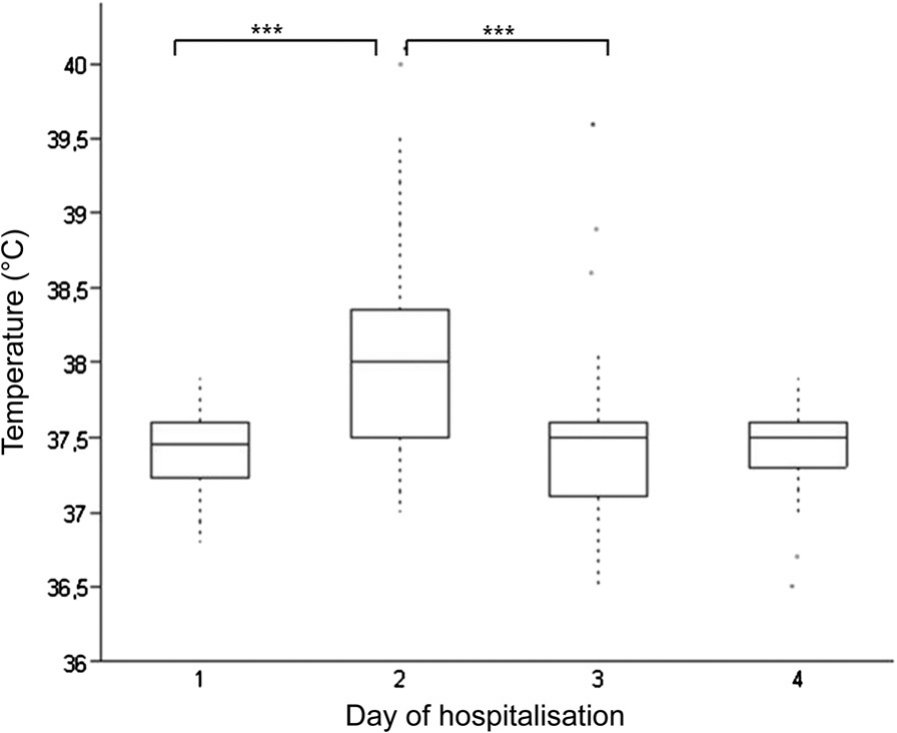
Daily rectal temperatures (in °C) measured in 27 horses before each injection (1) and on three consecutive days. There was a significant transient increase in the temperature on the first day after injection (Tukey-Kramer Multiple Comparison Test). The center horizontal line of the box plot marks the median of the sample. The edges of the box mark the first and third quartiles. The dotted lines define the 75th percentile plus 1.5 times the interquartile range (IQR) and the 25th percentile minus 1.5 times IQR. The singular dots represent the outside values. *** Significant difference (p ≤ 0.0001).

Based on the clinical examinations of the horses the vaccine was safe and well tolerated. The only consistent abnormal finding was a significant increase in body temperature on the day after injection (p < 0.00001, Figure 1). No difference in body temperature between the groups (p = 0.98) was observed. Hematology and blood biochemistry profile revealed no abnormalities at any time point. At the intradermal injection sites (3x27 injections = 81 injections in total), horses showed mild subcutaneous swelling (81/81 injections), reddening (10/81), exudation (7/81) and mild ulceration (2/81). These signs of acute inflammation occurred within the first three days after injection and resolved until the next treatment except in horse 11 were they persisted for 21 days after the first injection. Twentysix of 27 horses developed local dermal depigmentation restricted to the intradermal injection sites, only. The depigmentation was first observed after 22 days in 23 horses and after 81 days in 3 more horses. There were no signs of depigmentation observed in the monitored pigmented regions or melanoma lesions. No differences of local reactions were noted between treatment groups. No local reactions were observed at the sites of intramuscular injections.

### Caliper and ultrasonographic measurements of tumor size

**Figure 2:**
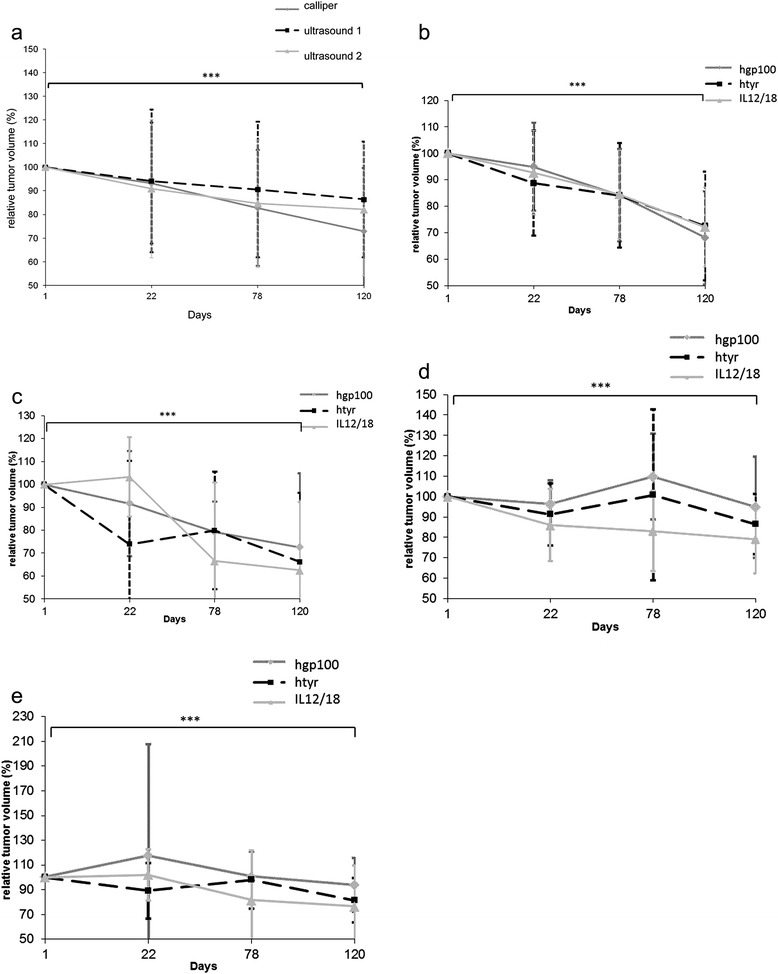
Relative tumor volumes of melanoma lesions during the observation period of 120 days. **a)**: Relative volumes (%) of locally and non-locally treated melanoma lesions calculated by caliper and ultrasound measurements (ultrasound examiner 1 and 2) on days 1, 22, 78 and 120. Relative volume of melanoma lesions decreased significantly from day 1 to 120 (Tukey-Kramer Multiple Comparisons Test). There were no statistically significant differences detected between the treatment groups, locally or non-locally treated melanoma lesions and measurement modality/ultrasound examiner. Dots, squares and triangles represent the median and the vertical lines the standard deviations. **b)**: Relative volumes (%) of non-locally treated melanoma lesions calculated by caliper measurements on days 0, 22, 78 and 120. Relative volume of all non-locally treated melanoma lesions decreased significantly on day 120. There were no statistically significant differences detected between the treatment groups. **2c)**: Relative volumes (%) of locally treated melanoma lesions calculated by caliper measurements on days 0, 22, 78 and 120. Relative volume of all locally treated melanoma lesions decreased significantly on day 120. There were no statistically significant differences detected between the treatment groups. **2d)**: Relative volumes (%) of non-locally treated melanoma lesions calculated by ultrasonographic measurements on days 0, 22, 78 and 120. Relative volume of all non-locally treated melanoma lesions decreased significantly on day 120. There were no statistically significant differences detected between the treatment groups. **2e)**: Relative volumes (%) of locally treated melanoma lesions calculated by ultrasonographic measurements on days 0, 22, 78 and 120. Relative volume of all locally treated melanoma lesions decreased significantly on day 120. There were no statistically significant differences detected between the treatment groups. *** Significant difference (p ≤ 0.0001).

In total 136 melanoma lesions (groups IL12/18: n = 42, hgp100: n = 50, htyr: n = 44) were measured. After stepwise elimination of insignificant variables and correlations the only variable influencing the relative volume was the examination day (p = 0.00001) and an individual effect of the horses. Post-hoc testing showed a significant decrease of the relative melanoma-volume over time. Mean relative volume of all measured melanoma decreased significantly to 71.5 ± 29.73% - equalling to 28.5% of tumor volume reduction - as measured by caliper and 87.0 ± 24.42% (13% tumor volume reduction), 82.1 ± 28.02% (17.9% tumor volume reduction) as measured by ultrasonography (ultrasound examiner 1, ultrasound examiner 2) (Figure 2, Table 3). The relative tumor volume ranged from 0 to 205% (caliper) and 0 to 194, 196% (ultrasound examiner 1, ultrasound examiner 2) at day 120.

**Table 3:** **Relative volume of melanoma lesions measured by calipers in individual horses on Day 120, and number of new lesions by Day 120**

**Treatment (encoded genes)**	**Horse ID**	**% of reduction of the tumor volumes at day 120 in reference to the volume on day 1**		**Number of new lesions**
		**Peritumoral treated melanoma lesion**	**Distant melanoma lesions**	
eqIL12, eqIL18	1	−16	−35,- 31,-30,-17, 2, 7, 10, 23	0
	2	−38	−40	0
	7	−33	−69, −7, −4	0
	9	2	−42, −24, −16	0
	12	−35	−78, −49, −46, −42, −26	0
	13	0	−39, −32	0
	17	−43	−40, −11, −8	0
	23	−47	**-**	0
	24	−26	−40, −21, −19, −12, −8, −4, −3, −3	0
eqIL12, eqIL18, hgp100	3	48	**-**	0
	5	−46	−56, −50, −36, 6	0
	6	−31	−75, −61, −40, −33, −6	0
	8	−59	−56, −49, −31, −31, −23, −15, 9, 22	0
	15	−46	−58, −43, −37, −23, −27, −21, 0, 0	0
	18	−16	−53, −41, −35, −25, −24, −23, −21, −20	0
	19	−43	−36	0
	22	−8	−47	0
	27	−45	−73, −45, −23, −15, −12, 0	0
eqIL12, eqIL18, htyr	4	−59	−79, −50, −32, −27, −24, −14, −12, 105	0
	10	−79	−65, −42, −32, −32, −22, −7, −5, 33	0
	11	−25	−58, −56, −46	0
	14	−71	−46, −32, −31	0
	16	−1	−33	0
	20	−20	−55, −46, −17, −15, −9, −5, −3, 12	0
	21	−38	**-**	0
	25	−18	−38, −30, −5, −15	0
	26	7	**-**	0

### Measurement of the humoral response induced by vaccination

No specific antibodies against htyr or hgp100 were detected in equine sera collected on days 1, 22, 78 and 120 (see Additional file 1: Appendix A, Additional file 2: Figure S1).

### Proof of function of DNA vectors: *In vitro* expression

The expression of all encoded genes was proven on mRNA level after transfection of cells *in vitro*. MIDGE-Th1 vectors were used in mixtures at weight ratios equal to those administered to the patients and complexed with SAINT-18. The amount of cDNA generated from mRNA was analysed in a RT-qPCR assay using a plasmid standard, allowing for detection and estimation of the mRNA expression levels produced from individual transgenes. eqIL12, eqIL18, htyr and hgp100 were shown to be expressed from all respective mixtures of MIDGE-Th1 vectors (Table 4).

**Table 4:** **Results of RT-qPCR to evaluate** ***in vitro*** **expression of transgenes from MIDGE-Th1 vectors encoding eqIL12, eqIL18, htyr and hgp100**

**MIDGE-Th1**	**Estimated mRNA / cDNA copy numbers per transgene (Ct values)**				
**vectors [μg]**	**eqIL12-p35**	**eqIL12-p40**	**eqIL18**	**htyr**	**hgp100**
eqIL12, eqIL18	1.14 + E03	1.52 + E04	3.16 + E03	< LOD	< LOD
[0.5 + 0.5]	(27)	(23)	(25)		
eqIL12, eqIL18, htyr	1.01 + E03	2.10 + E04	5.23 + E03	3.01 + E04	< LOD
[0.5 + 0.5 + 1.25]	(28)	(23)	(25)	(22)	
eqIL12, eqIL18,	9.10 + E02	9.79 + E03	1.98 + E03	< LOD	1.30 + E04
gp100	(28)	(24)	(26)		(23)
[0.5 + 0.5 + 1.25]					

LOD (limit of detection): = < 25 copies/reaction.

## Discussion

In the present clinical study, three treatments with mixtures of MIDGE-Th1 eqIL12 and ILRAP-eqIL18 alone or in combination with MIDGE-Th1 hgp100 or MIDGE-Th1 htyr, all complexed with the cationic transfection agent SAINT-18, resulted in a moderate reduction of the relative volume of examined melanoma lesions (28.5%). The lack of differences in treatment effects between the three groups suggests that MIDGE-Th1 hgp100 or MIDGE-Th1 htyr did not augment the effect of the other components of the treatment.

In former studies on equine melanoma the placebo group showed progression or no change in size during the study period of 64 days [28,31]. A placebo group was not included in this study because a positive anti-tumoral effect of treatment with interleukin-12 and −18 DNA had been demonstrated in these earlier studies. Ethical considerations regarding withholding any potentially effective treatment from client-owned horses precluded inclusion of an untreated cohort. However, it is reasonable to attribute the tumor size reduction observed in this study to the treatment, as no spontaneous regression of equine melanoma lesions has been reported up to date. Importantly, the size reduction was not only seen in locally treated but also in non-locally treated melanoma lesions indicating a systemic effect of the treatment. Comparing the development of volume of peritumorally treated and distant melanoma lesions, there was no statistically significant difference observed. The systemic effect might be based on a primarily unspecific immune response that, in the presence of melanoma antigens, converted into an anti-melanoma specific immune response. However, this is a hypothesis only since such aspecific immune response has not been detected in our assays.

The present study was the first to use a combination of DNA vectors encoding eqIL12 and eqIL18 for the therapy of equine melanoma. Previously either eqIL18 or eqIL12 DNA injected intratumorally induced some size reduction in equine melanoma lesions [28] but systemic effects were not evaluated. Experimental studies in mice [24,25] have proven synergistic effects of IL12 and IL18, suppressing collateral [26] or distant tumor growth [20].

In the present study the cationic-amphiphilic transfection agent SAINT-18 was used to enhance expression *in vivo*. SAINT-18 has been reported to strengthen the immune response against antigens encoded by MIDGE-Th1 vectors [30,32]. While the complexed DNA was very well tolerated in mice and rats [30], in the horses of the study presented here it caused local inflammation at the site of intradermal injection. This could be either explained by an inflammatory reaction triggered by the two expressed cytokines eqIL12 and eqIL18 upon transfection of dermal cells, or respectively and by a higher sensitivity of horses against DNA complexed with SAINT-18. In mice, expressed cytokines were found to mediate antitumoral effects by reduction of angiogenesis [20] as well as improved cellular immunity represented by increased IFN_γ_ production and enhanced cytotoxic T- and Natural Killer cell activities [25]. In addition to the local reaction, horses developed fever on the day post injection, a systemic reaction to the treatment. Dow et al. [33] showed that i.v. injections of plasmid DNA complexed with lipid-protamine with non-coding DNA, resulted in local inflammation of the lung and other organs of mice. Thus, it is possible that in the present study fever and the local inflammation were in part induced by the complexes of DNA and transfection agent. In mice injection of SAINT-18 formulated DNA was tolerated well. Neither local nor systemic signs of intolerance were observed [30]. The use of SAINT-18 in pigs proved to enhance their humoral immune response to DNA vaccination. The study did not mention adverse side effects of the transfection reagent [32]. However both, the fever as well as the signs of acute inflammation were transient and are therefore considered not to be a serious drawback regarding the safety of the vaccination. As Dow et al. [33,34] showed also that lipoplexes with DNA encoding IL12 induced a more effective antitumor response than lipoplexes with non-coding plasmid DNA in mice the effect of the DNA-SAINT-18-complex might have potentially enhanced the antitumoral effect of the Interleukins.

The tumor size reduction in the present study was moderate and – according to the Response Evaluation Criteria In Solid Tumors (RECIST) [35] or immune-related Response Criteria (irRC) [36] used in human medicine - would not be considered as tumor regression but as stable disease. However, Wolchok et al. consider stable disease as a positive therapeutic outcome in cancer immunotherapy [36].

Dosages of 100, 500 and 1500 μg plasmid DNA for hgp100 in humans [8] and for htyr in dogs [12] successfully induced detectable immune responses. In the studies of Lembcke et al. [17] and Phillips et al. [37] a dosage of only 100 μg plasmid induced a measurable, though variable and relatively weak, immune response in nontumor-bearing healthy horses. Thus the doses used in our study, i.e. 500 μg per antigen encoding MIDGE-Th1 vector per application should have been appropriate to induce a detectable immune response. Moreover, MIDGE-vectors are devoid of plasmid backbone DNA and therefore have a smaller molecule size, so their effective transgene dose per microgram DNA is much higher than that of conventional plasmids [30].

Tyrosinase and gp100 were found to be (over-)expressed in equine melanoma lesions [31,38,39]. Homology between equine and human proteins is comparable to the proteins of mice compared to human for gp100 [75.5% [16]] and canine tyrosinase as well as the mouse and human equivalent [84.4% and 87.5% [12]]. Since xenogenic DNA vaccination encoding these proteins in mice and dogs induced an antitumoral immune response [10-12,14,16] it can be assumed that the proteins chosen and the difference in the amino acid sequence are adequate.

The vaccination interval used in the recent study is comparable to immunisation strategies for infectious diseases and also to a therapeutic vaccination protocol against melanoma in humans [40]. However, vaccination against tumors generally elicits a weaker immunity than vaccination against infectious diseases [41]. In other studies, in which an immunological response was successfully induced, more frequent application schemes for therapeutical antitumor vaccines [8,12,13] or cytokine gene therapy were used [42]. Lembcke et al. [17] vaccinated healthy horses four times with human tyrosinase DNA every two weeks, resulting in the successful induction of specific antibodies and IFNγ producing cells. They stated that the altered immunologic status of tumor-bearing patients (tumor tolerance) might make it more challenging to induce a specific immune response to tumor antigens than in healthy animals [17].

No specific humoral response could be observed in the present study. In future studies additional tests to evaluate the cellular immune response [17] might give additional information about immunological mechanisms. However, in contrast to studies by Lembcke et al. [17] and Phillips et al. [37], we treated melanoma-bearing horses which might have developed an immune tolerance to gp100 and tyrosinase previously. To overcome this specific immune tolerance, higher DNA doses encoding xenogenic antigens and/or a more condensed immunisation schedule than in the present study might be necessary to induce a detectable immune response.

## Conclusions

In the present study, treatment of horses with MIDGE-Th1 vectors encoding eqIL12 and ILRAP-eqIL18 alone or in combination with MIDGE-Th1 vectors encoding hgp100 or human htyr, all complexed with the transfection agent SAINT-18 resulted in a measurable systemic antitumoral effect on equine melanoma lesions. To our knowledge this is the first report on a systemic antitumoral effect against equine melanoma upon treatment with DNA vectors.

Because no specific immune response was detected, it remains to be elucidated whether this systemic antitumoral effect was caused by interleukins expressed from the DNA vectors, or an unspecific immune reaction to the combination of DNA and transfection reagent.

In future studies, a stronger anti-tumoral effect as well as a detectable specific immune response may be induced by an increased vaccine dose and/or an improved dosing schedule. Additionally, more sensitive immune assays should be applied to characterize the immune response in detail.

## Additional files

Additional file 1:
**Supplemental Digital Content 1.** Contains supplementary text file 1. Additional file 2:
**Figure S1.** No specific antibodies were detected in serum samples from vaccinated horses on days 1, 22, 78 and 120.

## Abbreviations

ANOVA: Analysis of variance
DNA: Deoxyribonucleic acid
cDNA: complementary deoxyribonucleic acid
CHO: Chinese hamster ovary
EDTA: Ethylenediaminetetraacetic acid
eGFP: enhanced green fluorescent protein
eq: equine
FACS: Fluorescence-activates cell sorting
FCS: Fetal calf serum
gp: glycoprotein
hgp: human glycoprotein
htyr: human tyrosinase
i.d.: intradermally
i.m.: intramuscularly
IL: interleukin
ILRAP: IL-1beta receptor antagonist protein
irRC: immune-related Response Criteria
MIDGE: Minimalistic immunogenically defined gene expression
mRNA: messenger ribonucleic acid
PBS: Phosphate buffered saline
RECIST: Response evaluation criteria in solid tumors
RNA: Ribonucleic acid
RT-qPCR: Reverse transcription quantitative polymerase chain reaction
tyr: tyrosinase
